# Characterization of the antibacterial activity from ethanolic extracts of the botanical, *Larrea tridentata*

**DOI:** 10.1186/s12906-021-03344-9

**Published:** 2021-06-25

**Authors:** Tiffany Turner, Guillermo Ruiz, Johanne Gerstel, Jeffrey Langland

**Affiliations:** 1 Southwest College of Naturopathic Medicine, The Ric Scalzo Institute for Botanical Research, Tempe, AZ 85282 USA; 2 Arizona State University, Biodesign Institute, Tempe, AZ 85287 USA

**Keywords:** *Staphylococcus aureus*, Bacteria, Antimicrobials, Antibiotic, β-Lactam, Tincture, *Larrea tridentata*

## Abstract

**Background:**

β-lactam antibiotics are a class of broad-spectrum antibiotics consisting of all antibiotic agents that contain a β-lactam ring in their molecular structures. β-lactam antibiotics are only known to be isolated from fungi (e.g. *Acremonium chrysogenum, Penicillium chrysogenum* and *Aspergillus nidulans*) and bacteria (e.g. *Streptomyces clavuligerus*). We have shown that botanical extracts prepared from *Larrea tridentata* have strong antimicrobial activity against several bacteria, including members of *Staphylococcus* and *Streptococcus* genera.

**Methods:**

Through resistance studies, inhibitor assays, and ELISA testing, we demonstrated *L. tridentata* extracts may contain a β-lactam type antibiotic activity.

**Results:**

Based on the estimated β-lactam concentration within the extract, the antimicrobial activity of the *L. tridentata* extract was approximately 2000–8000-fold greater against *Staphylococcus* as compared to other β-lactams, penicillin or ampicillin. In the *L. tridentata* extract, this increased activity was found to be associated with the likely presence of a cofactor leading to increased potentiation of the β-lactam activity. This potentiation activity was also observed to enhance the activity of exogenously added natural penicillin antibiotics.

**Conclusions:**

Although constituents were not isolated in this study, the results obtained strongly support the presence of β-lactam type antibiotic activity and antibiotic potentiation activity present in ethanolic extracts prepared from *L. tridentata*.

## Background

Penicillin antibiotics are historically significant because they were the first drugs that were effective against many previously serious diseases such as tuberculosis, syphilis, and *Staphylococcus* infections. Not only was penicillin ground-breaking as a medicine in its own right, but the development of penicillin required vast advances in science and technology responsible for the development of all modern antibiotics and other medicinal bioproducts [1].

Since the discovery of penicillin from *Penicillium* in 1928, β-lactam antibiotics have continued to have a substantial influence on the world of medicine and have been isolated from both fungal and bacterial sources. Hydrophobic (with aromatic side chains) penicillins are only known to be produced by fungi, mainly *Penicillium chrysogenum* and *Aspergillus nidulans*, whereas hydrophilic cephalosporins are produced by both fungi (e.g. *Acremonium chrysogenum*) and bacteria (e.g. *Streptomyces clavuligerus*) [2]. These β-lactam compounds exert their antimicrobial effects by targeting penicillin binding proteins and by stimulating a reoccurring cycle of cell wall synthesis and breakdown leading to depletion of cellular resources and, ultimately, cell death [3].

In this research, we demonstrate the presence of β-lactam-like antibiotic activity from the botanical, *Larrea tridentata,* which may be derived either from the plant directly or potentially from endophytic microbes. Medicinal plant extracts have long been of interest as sources of novel antimicrobial agents and extracts from these botanicals have previously been shown to strongly inhibit the replication of multiple bacteria, including *Staphylococcus aureus* [4].

*Larrea tridentata*, also known as chaparral or creosote bush, is a shrubby plant which dominates some areas of the desert southwest in the United States and Northern Mexico. Previous research has already identified several lignans and flavonoids containing antibacterial and antimycobacterial activity within *Larrea tridentata* and *Larrea divaricata* [5, 6]. One of these antibacterial lignans is identified to be 3′-demethoxy-6-O-demethyl-isoguaiacin, whose novel target in methicillin-resistant *Staphylococcus aureus* is the ATP-binding cassette transport system proteins [7]. Additional research has also noted anti-helminthic and antiparasitic properties of *L. tridentata* [8, 9].

As bacterial resistance to antibiotics continues to claim thousands of lives in the United States each year, there is an urgent need to use existing antibiotics carefully, to find ways to reduce resistance and to discover novel antimicrobial agents [10]. The CDC reports that approximately 50% of prescribed antibiotics are either not necessary or not effective, and that the cost of antibiotic resistance ranges from $20 billion in direct costs to $55 billion per year when accounting for productivity losses [10]. Unfortunately, the rate of new antibiotic development is slow, and cannot keep up with the rising rate of antibiotic resistant infections [11]. Resistance to β-lactams can develop through any of the following: the production of β-lactamase enzymes, changes in penicillin binding proteins or the ability to bypass penicillin binding proteins, membrane impermeability to drugs, and efflux pumps [12].

It is suggested that to limit the development of antibiotic-resistant bacteria, antibiotics with multiple mechanisms should be used and care should be taken not to underdose potent antibiotics [13]. Therefore, in order to use antimicrobial plants appropriately, we must first understand their mechanism of action. Identifying the primary mechanism behind the antibacterial properties of botanicals holds several purposes. It enables the plants to be used more effectively as antibacterial agents, helps identify potential problems with use, and highlights a system of discovery that can be applied to other medicinal plants for uncovering additional novel antibacterial agents and their mechanisms. This research demonstrates the presence of antibacterial activity in botanical extracts prepared from *L. tridentata.* Although constituents were not isolated in this study, the results strongly support the likely presence of β-lactam type antibiotic activity and the presence of a potentiation constituent which enhances the β-lactam antimicrobial activity. These results support that this plant may hold promise for the isolation of potentially novel antibacterial compounds to help fight the rising problem of antibiotic resistant infections.

## Methods

### Chemicals and reagents

Tryptic soy broth and tryptic soy agar were obtained from Hardy Diagnostics (Santa Monica, CA). Antibiotics, Mueller Hinton agar, clavulanic acid, β-lactamase (from *Enterobacter cloacae*), and ethanol were obtained from Sigma-Aldrich Chemicals. The β-lactam ELISA kit (MaxSignal® β-Lactam ELISA Kit) was obtained from Bioo Scientific.

### Preparation of plant material

**Fig. 1:**
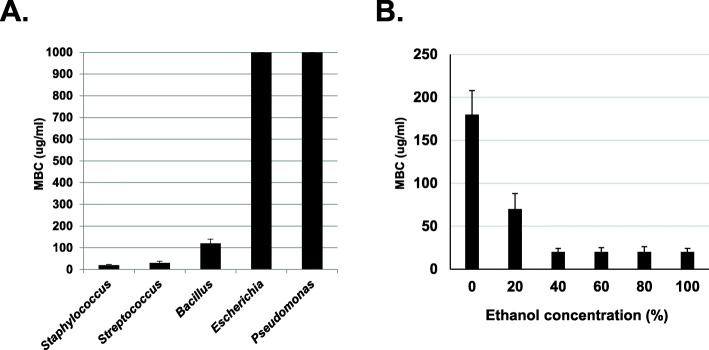
Antibacterial activity of *L. tridentata* extracts*.* Bacterial cultures were treated with increasing concentrations of the *L. tridentata* extract ranging from 1 to 1000 μg non-volatile constituents/ml media. The MBC was determined at 24 h. Part A was done with the indicated bacterial species using *L. tridentata* extracts prepared in 40% ethanol. Part B was done with *S. aureus* (ATCC 14775) using botanical extracts prepared with the indicated percentages of ethanol. Values shown with error bars represent the standard deviation from three separate experiments

*L. tridentata* plant material was obtained from Starwest Botanicals (Sacramento, CA) with high-performance thin-layer chromatography (HPTLC) performed to verify purity and authenticity (Starwest Botanicals Certificate of Analysis for Product Number 201255–51 and Product Lot number 57431). All plant material was subsequently verified by qualified botanical specialists using herbal pharmacopoeia monographs and reference keys. A voucher specimen of all plant material was deposited in a repository at the Southwest College of Naturopathic Medicine. 50 g *L. tridentata* dried leaves were ground to a fine powder using a high speed blender followed by resuspension in 40% ethanol (or ethanol concentrations indicated in Fig. 1B) at a ratio of 1:10 (dried plant material:extraction solution) and mixing by rotation at 60 rpm at room temperature for 24 h. The botanical debris was removed by centrifugation (3000×g for 10 min) and the supernatant sterilized by filtration through a 0.2 um filter. One ml of the final extract was dried to completion and the concentration of non-volatile constituents determined to be 37 mg/ml. For assays using the extracts, the treatment doses ranged from 1 to 1000 μg/ml based on the non-volatile constituents per ml. These doses are indicated in the figures and/or figure legends.

### Bacterial growth studies

Media and the bacterial cultures of antibiotic-sensitive *Staphylococcus aureus* (ATCC 14775), penicillin-resistant *Staphylococcus aureus* (ATCC 11632), multi-drug resistant *Staphylococcus aureus* (ATCC BAA-44), *Streptococcus pyogenes* (ATCC 12344), *Bacillus cereus* (ATCC 10876), *Escherichia coli* (ATCC 11229), and *Pseudomonas aeruginosa* (ATCC 35554) were obtained from Hardy Diagnostics (Santa Monica, CA). As described by ATCC, the *S. aureus* BAA-44 strain is resistant to a broad spectrum of antibiotics including ampicillin, amoxicillin/clavulanic acid, ciprofloxacin, cephalothin, doxycycline, gentamicin, erythromycin, imipenem, methicillin, penicillin, tetracycline, oxacillin, azithromycin, clindamycin, ceftriaxone, rifampin, amikacin and tobramycin. As described by ATCC, the *S. aureus* ATCC 11632 strain is resistant solely to β-lactam antibiotics including penicillin, ampicillin and methicillin.

For bacterial inhibition studies, minimum bactericidal concentrations (MBC) were determined. Minimum inhibitory concentrations (MIC) were not measured due to turbidity of the broth cultures which occurred following the addition of *L tridentata* extracts making reliable determination of MIC values difficult. Although MIC assays have been conducted in the past [14], for more accurate measurements, MBCs were determined. For MBC determination and growth studies, 18-h cultures (5 × 10^8^ colony-forming units/ml (CFU/mL)) were diluted into media (1:1000 dilution; tryptic soy broth (TSB)) followed by the addition of indicated concentrations of each botanical extract, antibiotic, or null control. The cultures were incubated at 37 °C with aeration (by continuous rotation) for 24 h. The CFU count was performed, after serial dilution and inoculation onto tryptic soy agar (TSA) plates and incubation for 24 h at 37 °C. The MBC was identified by determining the lowest concentration of the treatment that reduced the viability of the bacteria by ≥99.9%. For the *L. tridentata* extracts, the MBC was listed as the total concentration of non-volatile constituents present in the extract since the active constituent(s) has not been isolated or identified.

Antibiotic susceptibility testing was performed using the Kirby Bauer disc diffusion method according to the Clinical and Laboratory Standards Institute (CLSI: M100-S22) guidelines [15]. Bacterial suspensions were prepared by transferring 3–5 pure colonies into nutrient broth and adjusted to 0.5 McFarland standards. A sterile cotton swab was then dipped into the suspension and swabbed on surface of Mueller-Hinton agar plate. Standard antibiotic discs were placed aseptically and the inoculated Mueller Hinton agar plates were incubated at 37 °C for 16–18 h (penicillin 10 μg, tetracycline 30 μg, vancomycin 30 μg, and ciprofloxacin 5 μg). The diameters of the zones of complete inhibition were measured using calipers in mm.

### Resistance development

*S. aureus* (ATCC 14775) was used as a model organism to study the antibacterial activity the botanicals described in this study*.* A *S. aureus* strain resistant to the *L. tridentata* botanical extract was previously described and used in these studies [14]. As previously described, *S. aureus* (ATCC 14775) cultures (1 × 10 [6] CFU/ml) in TSB were treated with a 75% minimal inhibitory concentration (MIC) dose of the botanical extract [14]. The cultures were incubated at 37 °C with continuous aeration. Every 24 h, the bacterial culture was transferred to five different vials of fresh TSB media containing increasing amounts of the antimicrobial. The vial that demonstrated bacterial growth at the highest dose of antimicrobial was selected to continue the selection process. This process was repeated for a total of 15 days [14].

### Clavulanic acid assays

In order to evaluate the mechanism by which *S. aureus* had developed resistance to the *L. tridentata* extract [16], clavulanic acid (a β-lactamase inhibitor) was used. MBC assays were performed using the *L. tridentata* extract-resistant *S. aureus* [16] in the presence of increasing concentrations of the botanical extract along with a constant concentration of clavulanic acid (6 μg/ml).

### Treatment with β-lactamase and potentiation assay

*L. tridentata* extracts or penicillin stocks (1 mg/ml) were treated with 0.25 or 2.5 U/ml β-lactamase (from *Enterobacter cloacae*) for 1 h at 37 °C. The β-lactamase activity was then inactivated by incubation at 80 °C for 30 min. This extract was then used to measure the MBC of the treated penicillin and *L. tridentata* extracts. The effect of heating on the antimicrobial activity of the penicillin or the untreated *L. tridentata* extracts was tested by determining the MBC of the samples that were not treated with β-lactamase but heated for 80 °C for 30 min.

To test the ability of the *L. tridentata* extracts to enhance (potentiate) the activity of antibiotics, the β-lactam activity of the extract was removed/destroyed so that test antibiotics could then be added back to this treated extract. The β-lactam activity was removed as described above using 2.5 U/ml β-lactamase. Indicated individual antibiotics (Table 1) at varying concentrations were then added to this ‘β-lactam removed’ extract. The MBC value of these individual antibiotic containing extracts were determined and compared to the MBC value of the individual antibiotics alone.

**Table 1:**
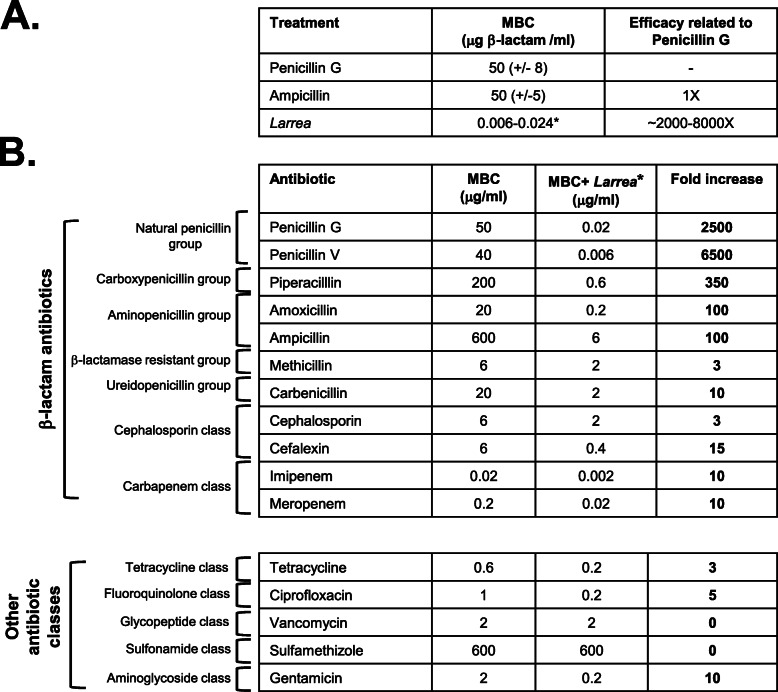
Enhancing/potentiation activity of *L. tridentata* extracts on individual antibiotics. Part A. MBC of the *L. tridentata* extract and penicillin/ampicillin based on β-lactam concentration. Bacterial cultures of non-antibiotic resistant *S. aureus* cultures (ATCC 14775) were treated with increasing concentrations of the indicated antibiotic (penicillin or ampicillin) or *L. tridentata* extract. The MBC was determined at 24 h and recorded based on the β-lactam concentration determined by ELISA. *For the *L. tridentata* extract, the MBC range is based on potential 25–100% cross reactivity between different classes of β-lactams for the ELISA assay. The efficacy relative to the MBC of penicillin is shown. Part B. MBC of individual antibiotics alone or in the presence of ‘β-lactam removed’ *L. tridentata* extracts.. *S. aureus* cultures (ATCC 14775) were treated with increasing concentrations of the indicated individual antibiotic or the individual antibiotic plus ‘β-lactam removed’ *L. tridentata* extract (*). The MBC of each sample was determined at 24 h and any fold increase in activity was determined by comparing the MBC of the antibiotic alone to the antibiotic with the ‘β-lactam removed’ *L. tridentata* extract

### ELISA assay

The *L. tridentata* botanical extract was tested for the presence of β-lactams using a commercially available ELISA kit (MaxSignal® β-Lactam ELISA Kit, Bioo Scientific) using the manufacturer’s recommended protocol.

## Results

To test the scope of antimicrobial activity of the *Larrea tridentata* extract, MBC assays were performed on a variety of bacterial genera. As shown in Fig. 1A, when bacterial cultures were treated with varying concentrations of a 40% ethanol extract of *L. tridentata*, inhibition in growth of *Staphylococcus aureus, Streptococcus pyogenes* and *Bacillus cereus* was observed at low concentrations of the extract (MBC values: *Staphylococcus aureus* at 20 μg/ml*, Streptococcus pyogenes* at 30 μg/ml, and *Bacillus cereus* at 120 μg/ml). Gram negative bacteria, including, *Escherichia coli* and *Pseudomonas aeruginosa* were resistant to treatment with this extract (MBC greater than at 1000 μg/ml). At the higher doses tested, 10 μl of the extract was added to 1 ml broth media resulting in a 1:100 or higher dilution of the extract. Control samples treated with similar doses of 40% ethanol alone (maximal final ethanol concentration of 0.4%) did not inhibit any bacterial growth (data not shown).

To test the optimal extraction method for these botanicals, extractions of *L. tridentata* were prepared with increasing percentages of ethanol and then tested for antimicrobial activity. As shown in Fig. 1B, extracts of *L. tridentata* were most active when extracted in 40% ethanol or higher. Again, at the higher doses tested, 10 μl of the extract was added to 1 ml broth media resulting in a 1:100 or higher dilution of the extract. Control samples treated with similar doses of ethanol ranging from 20 to 100% (maximal final ethanol concentration of 0.2–1%) did not inhibit any bacterial growth (data not shown).

**Fig. 2:**
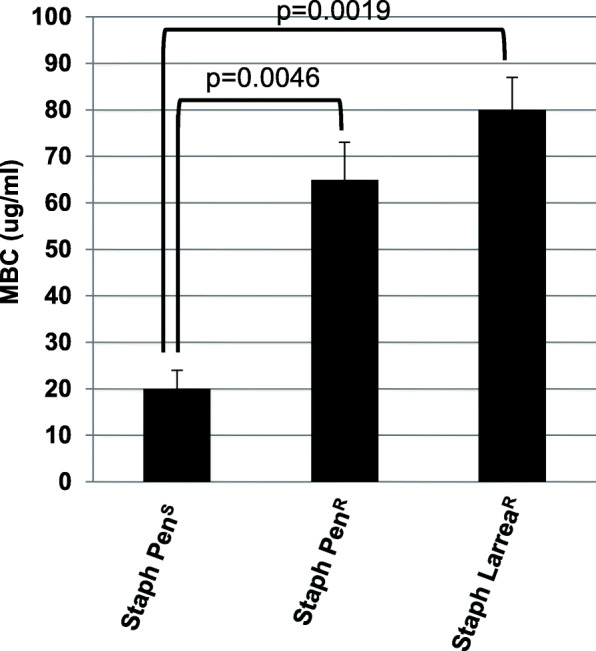
Activity of *L. tridentata* extract against botanical-resistant and penicillin-resistant strains of *S. aureus*. Bacterial cultures (Staph Pen^S^, ATCC 14775; Staph Pen^R^, ATCC 11632; and Staph Larrea^R^) were treated with increasing concentrations of the *L. tridentata* extract ranging from 1 to 100 μg non-volatile constituents/ml media . The MBC was determined at 24 h. Values shown with error bars represent the standard deviation from three separate experiments. Statistical analysis was performed using a paired t-test. Samples with statistically significant differences are indicated with the calculated *p*-value

*S. aureus* cultures resistant to the antibacterial activity of *L. tridentata* extracts were previous described [16]. The MBC of this *L. tridentata*-resistant *S. aureus* strain (Staph Larrea^R^) compared to the original *S. aureus* strain (ATCC 14775, Staph Pen^S^) is shown in Fig. 2. As shown, the Staph Larrea^R^ strain required an approximate 4-fold higher concentration of the extract to inhibit bacterial growth compared to the original Staph Pen^S^ strain (Fig. 2). When the antibacterial activity of the *L. tridentata* extract was tested against a commercially available β-lactam-resistant *S. aureus* strain (ATCC 11632, Staph Pen^R^), similar levels of resistance were observed where the Staph Pen^R^ strain required an approximate 3.25-fold higher concentration of extract to inhibit growth compared to the Staph Pen^S^ strain (Fig. 2). Though inconclusive, these results may suggest that the antibacterial activity of the *L. tridentata* extract was through a β-lactam type activity since the *S. aureus* strain (ATCC 11632) is only known to be resistant to β-lactam antibiotics.

**Fig. 3:**
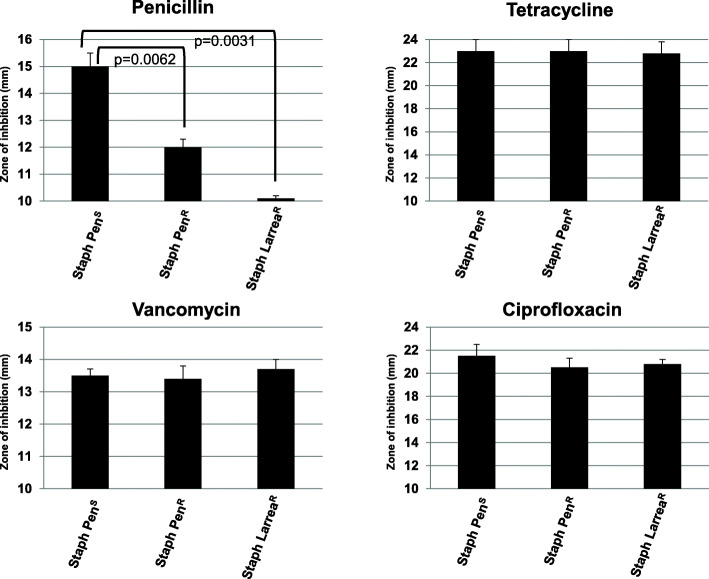
Spectrum of antibiotic resistance of botanical-resistant and penicillin-resistant *S. aureus* strains. Bacterial cultures (Staph Pen^S^, ATCC 14775; Staph Pen^R^, ATCC 11632; and Staph Larrea^R^) were tested for antibiotic susceptibility using the standard Kirby Bauer disc diffusion method. Standard antibiotic discs included penicillin 10 μg, tetracycline 30 μg, vancomycin 30 μg, and ciprofloxacin 5 μg. The diameters of the zones of complete inhibition were measured using calipers in mm. Values shown with error bars represent the standard deviation from three separate experiments. Statistical analysis was performed using a paired t-test. Samples with statistically significant differences are indicated with the calculated *p*-value

To test the spectrum of antibiotic resistance of the *L. tridentata*-resistant strain of *S. aureus*, this strain, the original *S. aureus* strain (ATCC 14775, Staph Pen^S^) and β-lactam-resistant *S. aureus* strain (ATCC 11632, Staph Pen^R^) were exposed to different antibiotics from different antibiotic classes using the Kirby Bauer disc diffusion method. As shown in Fig. 3, the Staph Larrea^R^ and the Staph Pen^R^ strains were both resistant to penicillin as indicated by the smaller zone of inhibition compared to the Staph Pen^S^ strain. This supports the results described in Fig. 2. When tested against tetracycline, vancomycin and ciprofloxacin, all three bacterial strains were found to be sensitive with no significant difference between the different strains (Fig. 3). Again, these results may support the presence of β-lactam type antibiotic activity present in the *L. tridentata* botanical extract since the *L. tridentata* resistant strain of *S. aureus* was only found to be co-resistant to penicillin for the antibiotics tested.

**Fig. 4:**
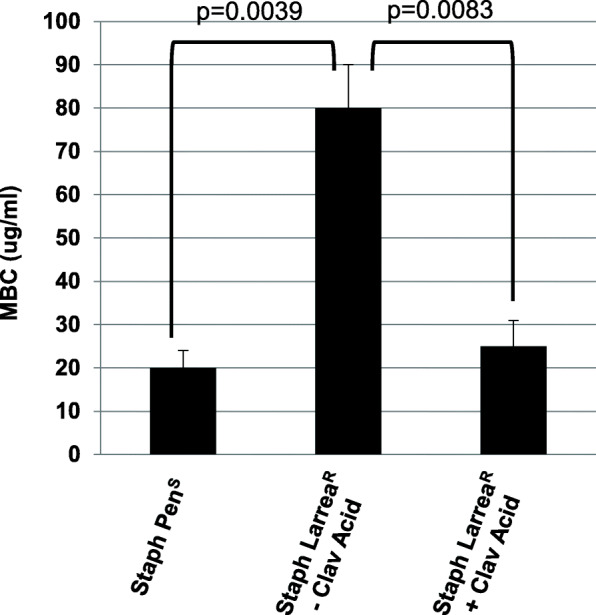
Effect of clavulanic acid on the growth of the botanical-resistant *S. aureus* strain. Bacterial cultures (Staph Pen^S^, ATCC 14775; and Staph Larrea^R^) were treated with increasing concentrations of the *L. tridentata* extract ranging from 1 to 1000 μg non-volatile constituents/ml media in the presence or absence of clavulanic acid (6 μg/ml). The MBC was determined at 24 h. Values shown with error bars represent the standard deviation from three separate experiments. Statistical analysis was performed using a paired t-test. Samples with statistically significant differences are indicated with the calculated p-value

Clavulanic acid is a known β-lactamase inhibitor of the Richmond types II, III, IV and V [14]. In order to test if the penicillin-resistance mechanism of action for the Staph Larrea^R^ strain was due to the presence of a β-lactamase, the Staph Larrea^R^ strain was treated with the *L. tridentata* extract in the presence and absence of clavulanic acid. As shown in Fig. 4, the antimicrobial sensitivity of the Staph Larrea^R^ strain to the *L. tridentata* extract was nearly fully restored when treated with the extract in the presence of clavulanic acid (compare the Staph Pen^S^ strain to the Staph Larrea^R^ strain plus clavulanic acid). Treatment of this strain with clavulanic acid alone (6 μg/ml) did not reduce replication (data not shown). The ability of clavulanic acid to inhibit the penicillin-resistance of the Staph Larrea^R^ strain suggests that this bacteria may have developed resistance to the antibacterial activity of *L. tridentata* extract through a β-lactamase mechanism, again supporting the potential presence of β-lactam type antibiotic activity present in the extracts of *L. tridentata.*

**Fig. 5:**
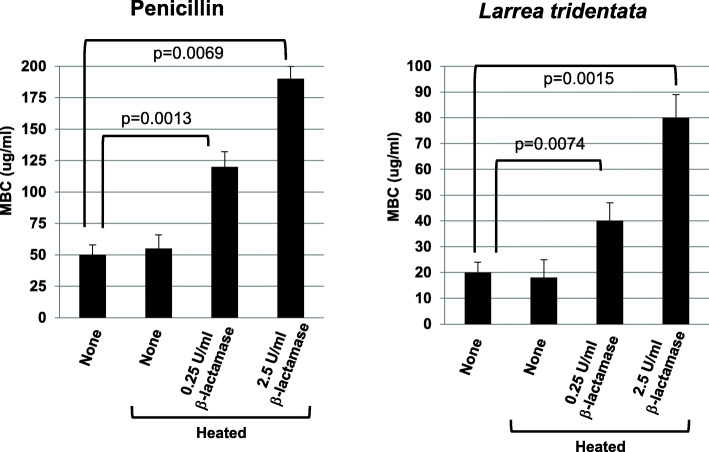
Effect of β-lactamase on the antibacterial activity of penicillin and *L. tridentata* extract. A penicillin stock solution or *L. tridentata* extract were treated with 0.25 or 2.5 U/ml β-lactamase followed by inactivation of the β-lactamase. Bacterial cultures of non-antibiotic resistant *S. aureus* (ATCC 14775) were treated with increasing concentrations of the treated or untreated penicillin stock solution (20–200 μg/ml) or *L. tridentata* extract (ranging from 1 to 1000 μg non-volatile constituents/ml media). The MBC determined at 24 h. Values shown with error bars represent the standard deviation from three separate experiments. Statistical analysis was performed using a paired t-test. Samples with statistically significant differences are indicated with the calculated p-value

To further support this hypothesis, *L. tridentata* extracts were treated with increasing concentrations of β-lactamase and then tested for antimicrobial activity against the non-antibiotic resistant strain of *S. aureus* (ATCC 14775). In this assay, a control penicillin solution or the *L. tridentata* extract were treated with the indicated concentrations of β-lactamase for 1 h followed by inactivation of the β-lactamase enzymatic activity by heating to 80 °C for 30 min. Inactivation of the β-lactamase activity was required for subsequent experiments. Heating of the penicillin solution or the *L. tridentata* extract did not inhibit the antibacterial activity (Fig. 5, compare untreated samples with and without heating). As shown in Fig. 5, β-lactamase treatment of both the *L. tridentata* extract and a control penicillin solution, significantly reduced the antibacterial activity against the non-antibiotic resistant strain of *S. aureus*. This further supports that the anti-*S. aureus* activity of the *L. tridentata* extract may be due to the presence of β-lactam type antibiotic activity present in the extract.

To further support the presence of a β-lactam activity in the *L. tridentata* extract, a β-lactam ELISA assay was performed. When the β-lactam ELISA assay was performed using extracts from *L. tridentata*, the presence of β-lactam was detected and the concentration relative to a penicillin standard was determined as approximately 3.2 ng β-lactam/ml extract (3.2 +/− 0.1 ng/ml). The ELISA kit used was able to detect a wide range of β-lactam type antibiotics, although to varying degrees, with cross-reactivity varying from 25 to 100% depending on the type of β-lactam present (based on manufacturer’s analysis). Since this assay was done in comparison to a penicillin standard (which is most highly detected by the ELISA kit), the concentration of the β-lactam could be up to 4-fold higher than that shown, theoretically ranging from 3.2–12.8 ng β-lactam/ml of extract based on the manufacturer’s cross reactivity analysis.

Based on the concentration of the β-lactam measured by the ELISA assay in the *L. tridentata* extract, the antibacterial activity of these extracts could be directly compared to that of known β-lactams, including penicillin and ampicillin. The MBC of penicillin, ampicillin and the *L. tridentata* extract based on the ELISA β-lactam concentrations is shown in Table 1A. Values for the *L. tridentata* extract is listed as a range based on the 25–100% cross reactivity of the ELISA assay. Based on the β-lactam concentrations, the *L. tridentata* extracts were approximately 2000–8000 times more active than the penicillin or ampicillin antibiotics (Table 1A).

The increase in relative antibacterial activity observed for the *L. tridentata* extract could potentially be due to a more active β-lactam constituent or the presence of a second constituent which enhances or potentiates the β-lactam activity. To test for enhancing/potentiation activity, the *L. tridentata* extract was treated with β-lactamase to remove the β-lactam activity from the extract (followed by heating to destroy the activity of the β-lactamase enzyme). As described in Fig. 5, heating alone did not alter the antibacterial activity of the extract and the β-lactamase treated extracts was tested and confirmed to have no anti-*S. aureus* (ATCC 14775) activity at the doses tested (data not shown). This ‘β-lactam removed’ *L. tridentata* extract was then combined with increasing concentrations of single, individual standard antibiotics and the MBC of each individual antibiotic alone was compared to the MBC of each individual antibiotic plus the ‘β-lactam removed’ *L. tridentata* extract. Any increase in activity of the antibiotic in the presence of the ‘β-lactam removed’ *L. tridentata* extract was recorded. As shown in Table 1B, the addition of the ‘β-lactam removed’ *L. tridentata* extract to penicillin G and penicillin V individually, was able to increase the activity of each of these antibiotics 2500 to 6500-fold, respectively. These were values similar to the increase in the relative activity of the *L. tridentata* extract compared to penicillin or ampicillin based on the β-lactam concentration (Table 1A). The addition of the ‘β-lactam removed’ *L. tridentata* extract to individual antibiotics belonging to the aminopenicillin group or carboxypenicillin group, increased the activity of these individual antibiotics 100–350-fold (Table 1B). Only minor increases in activity (3–15-fold) were observed when the ‘β-lactam removed’ *L. tridentata* extract was added to individual antibiotics belonging to the other groups of β-lactam antibiotics (Table 1B, see specific antibiotics in the β-lactamase resistant group, ureidopenicillin group, cephalosporin group, and carbapenem group). When the ‘β-lactam removed’ *L. tridentata* extract was added to individual non-β-lactam antibiotics, no or only minor increases in activity were observed (Table 1B, see tetracycline, ciprofloxacin, vancomycin, sulfamethizole, and gentamycin). All the aforementioned antibiotic assays were done in the presence of 40% ethanol alone (extraction vehicle) with no observable effect on individual antibiotic antibacterial activity (data not shown). These results likely suggest the presence of a specific β-lactam enhancing or potentiation activity present in the *L. tridentata* extract which was able to specifically increase the β-lactam antibiotic antibacterial activity of natural penicillin group antibiotics several thousand-fold and aminopenicillin and carboxypenicillin antibiotics several hundred-fold.

**Fig. 6:**
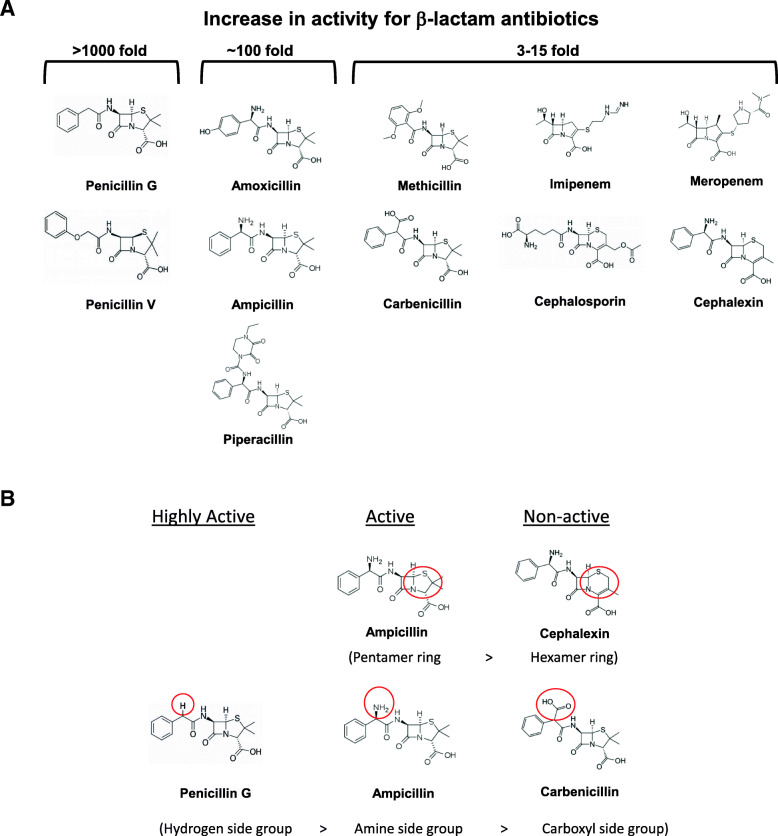
Structural characteristics of β-lactam antibiotics related to *L. tridentata* extract potentiation effects. A) The molecular structures of β-lactam antibiotics separated based on levels of *L. tridentata* extract potentiation effects. B) Required structural features for *L. tridentata* extract potentiation effects on β-lactam antibiotics

From the results observed in Table 1B, the structural requirements for the specific β-lactam antibiotics onto which the *L. tridentata* antibacterial enhancing/potentiation requires can be elucidated. The activity of ampicillin was able to be enhanced/potentiated by the *L. tridentata* extract whereas cephalexin was not. These compounds have similar structures except for a pentamer ring attached to the β-lactam ring for ampicillin and a hexamer ring attached to the β-lactam ring for cephalexin (Fig. 6A and B). This pentamer ring is present in all the β-lactam compounds which were able to be potentiated for activity suggesting a necessity for this structure. In addition, the structure of the side groups was important for potentiation where penicillin G, ampicillin, and carbenicillin have similar structures except for the side group shown in Fig. 6B. The most highly *L. tridentata* extract enhanced/potentiated β-lactam antibiotics had a hydrogen side group (> 1000 fold), while the moderately potentiated β-lactam antibiotics had an amine side group (~ 100 fold), and the non-potentiated β-lactam antibiotics had a carboxyl side group (Fig. 6B).

To test if the β-lactam antibacterial enhancing/potentiation activity present in the *L. tridentata* extract could increase the activity of penicillin towards antibiotic resistant strains of *S. aureus*, the ‘β-lactam removed’ *L. tridentata* extract was combined with increasing concentrations of penicillin G and the MBC determined against 3 different strains of *S. aureus*. As shown in Table 2, the *L. tridentata* potentiation extract increased the activity of penicillin G against non-antibiotic resistant *S. aureus* (ATCC 14775) approximately 1000-fold (similar to that observed in Table 1B). When tested against the penicillin-resistant strain of *S. aureus* (ATCC 11632), a moderate increase in activity was observed of approximately 100-fold suggesting increased sensitivity of this strain to the antibiotic in the presence of the potentiation constituent (Table 2). When tested against a multi-drug resistant strain of *S. aureus* (ATCC BAA-44), no significant increase in activity to the antibiotic was observed (Table 2).

**Table 2:**
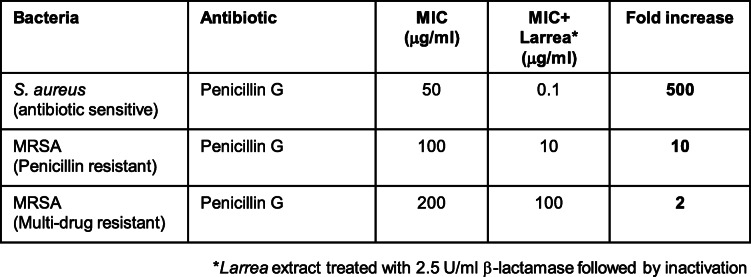
Antimicrobial activity penicillin or penicillin plus *L.tridentata* potentiation/enhancing activity against antibiotic-resistant *S. aureus* strains.. Bacterial cultures (antibiotic sensitive *S. aureus* (ATCC 14775), penicillin-resistant *S. aureus* (ATCC 11632), and multi-drug resistant *S. aureus* (ATCC BAA-44)) were treated with increasing concentrations of penicillin or penicillin plus ‘β-lactam removed’ *L. tridentata* extract (*). The MBC was determined at 24 h. Any fold increase in antimicrobial activity between penicillin alone compared to penicillin plus ‘β-lactam removed’ is noted

## Discussion

Although constituents were not isolated in this study, the results strongly support the presence of β-lactam type antibiotic activity and antibiotic potentiation activity in ethanolic extracts prepared from *L. tridentata*. The results suggest the presence of β-lactam type antibiotic activity in a 40% ethanol *L. tridentata* extract that is responsible for the antibacterial activity observed. Although not one assay performed would confirm this, together the 1) co-resistance of the β-lactam-resistant *S. aureus* strain to the *L. tridentata* extract, 2) the inhibition of resistance of a *L. tridentata*-resistant *S. aureus* strain by the addition of clavulanic acid, 3) the inhibition of antibacterial activity of the *L. tridentata* extract by the addition β-lactamase, and 4) the detection of a β-lactam constituent in the *L. tridentata* extract by ELISA assay strongly support the presence of β-lactam antibiotic activity in the *L. tridentata* extract.

Many bacteria that develop resistance to β-lactam antibiotics do so by the production of a β-lactamase that cleaves the β-lactam ring thereby deactivating the molecule’s antibacterial properties. Clavulanic acid is a drug that functions as a β-lactamase inhibitor. The ability of clavulanic acid to restore the antimicrobial activity of the *L. tridentata* extract against the *L. tridentata* resistant strain supports that this bacteria likely developed resistance through the expression of a β-lactamase enzyme and supports that the antibacterial activity of this botanical occurred through β-lactam-type activity. Further experiments using β-lactamase in combination with the botanical extract supported this as adding a β-lactamase directly to the extracts reduced their antimicrobial activity. In addition, the ELISA for β-lactams further supported the presence of a β-lactam antibiotic in the *L. tridentata* extract.

Historically, β-lactams have been isolated from fungi and bacteria. This study demonstrated the presence of β-lactam type antibiotic activity in a *L. tridentata* botanical extract. Although, β-lactams have not been isolated from plants, the discovery of conjugate β-lactams, where the β-lactam nucleus is N-linked to a terpenoid, have been discovered and marks the apparent ability of higher plants for produce β-lactams [17]. In addition, we cannot rule out the possibility of this β-lactam molecule being synthesized by endophytic microbes present on or within the plant. Although our extracts were prepared from dried plant material, potential constituents synthesized by endophytes may still be present. Notably, we have prepared and tested *L. tridentata* extracts from multiple geographic sources as well as common sources from multiple times throughout the year (over 15 uniquely distinct samples) and consistently found the β-lactam activity described in this manuscript in all the samples tested (data not shown). Again, this does not confirm whether this β-lactam activity was synthesized by the plant itself or by an endophytic microbe, but if made by an endophytic microbe, it is likely an organism commonly, or even symbiotically, associated with *L. tridentata*.

Although we have not yet isolated a β-lactam molecule from *L. tridentata*, extracts from *L. tridentata* had approximately 2000-8000-fold greater activity, respectively, against a non-antibiotic resistant strain of *S. aureus* as compared to penicillin or ampicillin (based on β-lactam concentrations). For the *L. tridentata* extract, this increased activity was found to be likely associated with an additional β-lactam enhancing/potentiation activity present in the extract. Previous studies using extracts of *Arctostaphylos uva-ursi* identified a compound, corilagin, which was shown to reduce the MIC of oxacillin, a β-lactam, against methicillin-resistant *S. aureus* via its inhibitory activities on PBP2’ and its inhibition of β-lactamase [18, 19]. In the present study, our results on *L. tridentata* suggests the presence of similar β-lactam enhancing/potentiation activity that could dramatically increase the activity of natural penicillin antibiotics as well as specific β-lactams within the aminopenicillin or carboxypenicillin groups. This suggests specificity of this potentiation activity related to the structural features and side-groups of the β-lactam molecule.

## Conclusions

In combating bacterial antibiotic resistance, conventional β-lactamase inhibitors, such as clavulanate, sulbactam, and tazobactam, have already been shown to play an important role [13]. The presence of potentially a novel β-lactam compound from *L. tridentata* extracts as well as a compound to enhance the activity of specific β-lactam antibiotics may provide additional resources in the fight against bacterial infections. Once isolated, the antibacterial β-lactam and β-lactam enhancing/potentiation compounds could potentially be used together or combined with existing drugs to form new solutions for antibiotic-resistant infections, as has been suggested elsewhere to help avoid drug resistance [12].

When using medicinal antimicrobial plants, knowing the mechanism allows physicians to potentially combine plants with different mechanisms of action in order to target an infection synergistically and decrease the risk of the bacteria becoming resistant. Furthermore, isolating the active compounds and determining the best methods for extraction and standardization will help to limit the problem of underdosing and potentially allowing a resistant infection to develop. In addition, it is imperative to highlight the presence of such compounds within medicinal botanicals to prevent misuse of these botanicals and to stem potential harm from assumptions that botanicals can be used without potential risks.

Further studies will be undertaken to isolate and characterize the active constituents in the *L. tridentata* botanical extracts. Antimicrobial botanical characterization will help our understanding of botanical extracts and could lead to the development of novel antibiotics at a time when antibiotic-resistance is becoming a major issue in the healthcare industry.

## Data Availability

No additional information is supplied as a supplementary file. Additional questions or information may be obtained by contact the Corresponding author, Jeffrey Langland.

## Abbreviations

HPTLC: high-performance thin-layer chromatography
TSB: tryptic soy broth
TSA: tryptic soy agar
MBC: minimum bactericidal concentration
MIC: minimum inhibitory concentration
ELISA: enzyme linked immunosorbent assay
CFU: colony forming units
